# A study on the relationship between mouth breathing and facial morphological pattern

**DOI:** 10.1016/S1808-8694(15)30101-4

**Published:** 2015-10-19

**Authors:** Ana Paula Bianchini, Zelita Caldeira Ferreira Guedes, Marilena Manno Vieira

**Affiliations:** a Master’s degree, speech therapist.; b Doctoral degree, assistant professor of Human Communication Disorders, Speech Therapy Department, Sao Paulo Federal University.; c Doctoral degree, assistant professor of Human Communication Disorders, Speech Therapy Department, Sao Paulo Federal University.

**Keywords:** mouth breathing, face, anthropometry

## Abstract

Breathing is responsible for facial and cranial morphology development.

**Aim:**

investigate in order to see if there is any relationship between oral breathing and facial type.

**Material and Methods:**

119 male and female teenagers, with ages ranging between 15 and 18 years. The sample was separated in two groups: A-50 teenage oral breathers, 28 males and 22 females; and group B- 69 teenage nasal breathers, 37 males and 32 females. The sample was collected at the Centro de Atendimento e Apoio ao Adolescente do Departamento de Pediatria da UNIFESP/ EPM. We evaluated breathing and facial measures.

**Results:**

by means of anthropometric indexes we classified facial types and associated them with the person’s breathing type, Hypereuriprosopic (Total=0; oral breathers 0%; nasal breathers 0%; Euriprosopic (Total=14; oral breathers 2.52%, nasal breathers 9.24%;Mesoprosope (Total=20; oral breathers 19.32%; nasal breathers 21.01%, Leptoprosopic (Total=37; oral breathers 14.29%; nasal breathers 16.81%; Hyperleptoprosopic (Total =48; oral breathers 5.89% nasal breathers 10.92%). The mesoprosopic facial type was found in 48 teenagers (40.33%) of whom 25 (21.01%) were oral breathers and 23 (19.32%) were nasal breathers. Conclusion: it was not possible to prove the existence of an association between oral breathing and facial type.

## INTRODUCTION

Normal breathing is nasal, making it possible for inhaled air to be purified, filtered, warmed and humidified before reaching the lungs. Nose breathing protects the upper airways and is responsible for adequate craniofacial development.

Mouth breathing may result from upper airway obstruction or from habit wherein air flows through the mouth. According to the literature, this form of breathing may change the growth pattern of the face and lead to morphological and functional alterations in the whole organism.

Certain authors, such as Moccelin & Ciuff (1997)^1^, Marchesan (1998)^2^, Lusvarghi (1999)^3^ and Di Francesco (1999),^4^ have defined mouth breathers as those persons with half-open, dry and cracked lips, an anteriorized tongue, weak mandibular elevator muscles, a deep and narrow palate, dental alterations and predominantly vertical face growth.

Altered facial growth in mouth breathers has been studied by various health care professionals, including medical doctors, speech therapists and orthodontists.

Anthropometry is that part of anthropology that investigates the body measurements. Cephalometrics is that part of anthropometry that studies the linear measurements and angles of the head.

Indices are used in anthropology to describe sizes regardless of absolute values. The facial morphological index is the centesimal ratio between the morphological height and width. This index classifies faces as leptoprosopic - long and narrow face; euryprosopic - wide and short face; and mesoprosopic - balanced facial width and height. (Avila, 1958^5^).

The aim of this study was to verify a possible relation between mouth breathing and the facial type, to ascertain the validity of statements in the literature describing mouth breathers as having a long face.

## MATERIAL AND METHOD

We assessed 119 male and female adolescents aged between 15 and 18 years. The sample was divided into two groups: group A - 50 mouth-breathing adolescents, 28 male and 22 female, and group B - 69 nose-breathing adolescents, 37 male and 32 female. The sample was obtained from the Adolescent Clinic and Support Center of the Pediatrics Department, UNIFESP/ EPM.

Adolescents that had genetic syndromes, malformation of the mouth and face, mental disability, cranial trauma, mutilation and dental anomalies of number and or size were excluded from the study.

The type of dental occlusion was not taken into account.

The mouth-breathing group contained adolescents that had mixed or mouth breathing.

Subjects underwent phonoaudiological assessments that consisted of the following steps:

I) a clinical history
II) a clinical phonoaudiological test.
I) Clinical history:
  The clinical history was taken from the participant and/or the caretaker to obtain identification data (name, age, sex, race) and information about respiratory conditions such as rhinitis, sinusitis, asthma, bronchitis, frequent colds, tonsillitis, daytime and/or nighttime mouth breathing and nighttime snoring.
II) Clinical phonoaudiological test:

The clinical test was composed of the following steps:

A - Assessment of breathing.
  Based on direct observation of the resting position and on tests.
A1 - Lip position of rest, classified as open or closed. Open was when the patient’s lips did not touch and when muscular effort was required for contact between the upper and lower lip, a position that was not maintained for long.
A2 - Breathing was also assessed by filling the mouth with water; if the patient was unable to keep the mouth closed for at least three minutes, he or she would be considered a mouth breather. This test was supplemented by observation made during other clinical tests and by information obtained directly from the patients. (Vieira, 1986^6^).
A3 - An Altman’s nasal breath mirror (in millimeters), placed below the nostrils, was used to assess the presence and symmetry of nasal airflow. Patients were instructed to breathe normally for a few seconds, after blowing their noses.

### B) Assessment of the face

The facial index, the relation between facial height and width, was calculated to find the facial type. Measurements were taken directly from the face of each adolescent.

Subjects were seated with their hips, knees and ankles at 90° for the clinical test. Feet were flat on the ground, the spine was erect and touching the back of the chair. The head was oriented according to Frankfurt’s plane, parallel to the horizontal plane and with its median sagittal plane perpendicular to the horizontal plane.

The facial height, or the length of the anatomical face, was measured as a straight line from the nasion to the gnathion. The nasion is the cephalometric point on the midline of the face that is located on the nasofrontal suture. The gnathion is the most anterior and inferior point on the suture of the mentum along the midline of the face; it may be palpated in living persons.

The facial width, the distance between the most laterally projecting points of the zygomatic arch, was measured between the right and left zygions. It may be established in living persons.

Facial height and width were measured in millimeters using a Mitutoyo model 500143 B digital pachymeter to which a 9 cm and 10 cm metal piece was attached on the external tips to make it possible for the rods to measure the facial height and width (zygions).

The facial proportion was obtained from the facial index or morphological facial index, which was based on these measurements. The facial index or morphological facial index is the centesimal relation between the height and width of the face, as follows:

$\begin{array}{ll} \text{Facial}\;\text{Morphological}\;\text{Index}= & \frac{\text{Facial}\;\text{height}\;\;\;\;\;\;\;\;\;\times 100}{\text{Bizygomatic}\;\text{diameter}} \end{array}$

This index is used to classify the facial types, as follows: hypereuryprosopic - X-78.9; euryprosopic - 79.0-83.9; mesoprosopic - 84.0-87.9; leptoprosopic - 88.0-92.9; hyperleptoprosopic - 93.0-X. (Avila, 19585).

The UNIFEST/EPM Research Ethics Committee approved this study (protocol number 0738/03) and subjects signed a free informed consent form.

## RESULTS

The hypereuryprosopic facial type was not found in our facial frequency study of male and female mouth and nose breathing groups. The most frequent facial type in males was the hyperleptoprosopic face, found in 33 adolescents (27.73%). In females, the most frequent facial type was the leptoprosopic face, found in 16 adolescents (13.45%). This difference is statistically significant, at p=0.008 (p< 0.05) (Table 1).

**Table 1:** Facial type’s frequence of occurrence in nasal and oral breathing males and females.

Gender						
Face	Female		Male		Total	
	Nº	%	Nº	%	Nº	%
Hypereuriprosopic	0	0	0	0	0	0
Euriprosopic	11	9,24	3	2,52	15	1,76
Mesoprosopic	12	10,08	8	6,73	20	16,80
Leptoprosopic	16	13,45	21	17,65	37	31,10
Hyperleptoprosopic	15	12,61	33	27,73	48	40,34

Total	54	45,38	65	54,62	119	100

The relation between facial types and the type of breathing revealed that the mesoprosopic face was the most frequent facial type, found in 25 mouth breathers (21.01%) and in 23 nose breathers (19.32%) (Table 2).

**Table 2:** Facial types frequence of occurence in oral and nasal breathing.

Breathing						
Face	Nasal		Oral		Total	
	Nº	%	Nº	%	Nº	%
Hypereuriprosopic	0	0	0	0	0	0
Euriprosopic	11	9,24	3	2,52	14	11,76
Mesoprosopic	25	21,01	23	19,32	48	40,33
Leptoprosopic	20	16,81	17	14,29	37	31,10
Hyperleptoprosopic	13	10,92	7	5,89	20	16,81

Total	69	57,98	50	42,02	119	100

The relation between facial types and mouth and nose breathing in females showed that the leptoprosopic face was the most frequent facial type in nose breathers (10 subjects - 18.52%) and the hyperleptoprosopic face was the most frequent facial type in mouth breathers (9 - 16.66%) (Table 3).

**Table 3:** Facial type’s frequence of occurence in oral and nasal breathing in females.

Breathing						
Face	Nasal		Oral		Total	
	Nº	%	Nº	%	Nº	%
Hypereuriprosopic	0	0	0	0	0	0
Euriprosopic	8	14,81	3	5,55	11	20,37
Mesoprosopic	8	14,81	4	7,4	12	22,23
Leptoprosopic	10	18,52	6	11,11	16	29,62
Hyperleptoprosopic	6	11,12	9	16,66	15	27,78

Total	32	59,26	22	40,74	54	100

The relation between facial types and mouth and nose breathing in males showed that the hyperleptoprosopic face was the most frequent facial type in both nose breathers (19 adolescents - 29.23%) and mouth breathers (14 adolescents - 21.53%) (Table 4).

**Table 4:** Facial type’s frequence of occurence in oral and nasal breathing in males.

Breathing						
Face	Nasal		Oral		Total	
	Nº	%	Nº	%	Nº	%
Hypereuriprosopic	0	0	0	0	0	0
Euriprosopic	3	4,61	0	0	3	4,61
Mesoprosopic	5	7,70	3	4,61	8	12,31
Leptoprosopic	10	15,39	11	16,93	21	32,31
Hyperleptoprosopic	19	29,23	14	21,53	33	50,77

Total	37	56,93	28	43,07	65	100

A comparative analysis between mouth and nose breathing groups based on Dunnett’s test7 found no predominance of one facial type over any other (Table 5).

**Table 5:** Comparative analysis between groups A and B as far as facial type classification according to gender is concerned.

Breathing										
Face	Nasal				Oral					
	Female		Male		Female		Male		Total	
	Nº	%	Nº	%	Nº	%	Nº	%	Nº	%
Hypereuriprosopic	0	0	0	0	0	0	0	0	0	0
Euriprosopic	8	14,8	3	4,6	3	5,6	0	0	14	11,8
Mesoprosopic	8	14,80	5	7,70	4	7,40	3	4,60	20	16,80
Leptoprosopic	10	18,5	10	15,4	6	11,1	11	16,9	37	31,1
Hyperleptoprosopic	6	11,1	19	29,2	9	16,7	14	21,5	48	40,3

Total	32	59,3	37	56,93	37	40,7	28	43	119	100

## COMMENTS

The investigation of facial types in mouth and nose breathing males and females showed that there were 33 male hyperleptoprosopic adolescents (27.73%) and 16 female leptoprosopic adolescents (13.45%). The X2 test revealed that there was a statistically significant difference between sexes (Table 1).

Our results are similar to those published by Gross, Kellum Hale et al. (1989).^8^ In their comparison between females and males they found the long face in 38.5% of their male subjects and in 30.9% of their females subjects out of a sample containing 133 subjects.

The study of facial types in mouth breathers showed that the mesoprosopic face was the most frequent facial type in 23 adolescents (19,32%) (Table 2).

These findings are similar to those of Ferreira (1999),^9^ who also found that a higher proportion of his mouth-breathing subjects had the mesoprosopic face (5 patients - 44%). Our findings are also similar to those of Sabatosk & Maruo (2002)^10^ and Aidar, who “found 18 mesoprosopic facial types in a study of mouth breathers (40.90%)” (personal communication*).

Our results disagree with those of Mattar (2002)^11^ who found a correlation between facial morphology and the type of breathing; in this study, mouth breathers (84.68% subjects) were predominantly dolichofacial and nose breathers were mostly mesofacial (88.07 subjects).

Our results also disagree with those of Harterink & Vig (1989),^12^ Fields,^13^ Mocellim & Ciuff (1997),^1^ and Manganello (2002),^14^ who state that the mouth-breathing habit could change the face and muscle balance, resulting in a higher rate of the long face (*Aidar L, Mota J, Marins C. 2004, personal communication).

Investigation of the face type in nose breathers revealed that there were 25 mesoprosopic adolescents (21.01%),^20^ leptoprosopic adolescents (16.81%), 13 hyperleptoprosopic adolescents (10.92%) and 11 euryprosopic adolescents (9,24%) (Table 2).

These results are similar to those of Jabur (1997),^15^ who found a balance in the distribution of patients with vertical growth (9 subjects - 39.13%) and patients with a harmonic pattern (10 subjects - 43.47%). Our results also agree with those of Vig, Sarver, Hall and Warren (1981),^16^ who found normal facial proportions and labial competence in 10 subjects (35.71%).

A higher frequency of the mesoprosopic facial type in mouth breathers was not expected; in the literature many authors, such as Tourne (1990)^17^ and Mocellin & Ciuff (1997),^1^ have stated that there is a relation between the long face and mouth breathing. According to these authors, mouth breathers keep their mouths constantly open and the tongue in a lowered position, without exerting pressure on the palate, resulting in external compression of the maxilla by the external muscles of the mouth. The hard palate tends to deepen, forming an ogival palate. The palate puts upward and forward pressure on the cartilaginous septum, causing its deviation and the elongated and narrowed face.

Investigation of the face type frequency in female nose and mouth breathers revealed that there were 10 leptoprosopic adolescents (18.52%) and 9 hyperleptoprosopic adolescents (16.66%) (Table 3).

In males, the hyperleptoprosopic face was the most frequent facial type in both nose breathers (19 adolescents - 29.23%) and mouth breathers (14 adolescents - 21.53%) (Table 4).

We were unable to compare these results with the literature, as we found no published papers describing the frequency of facial types in adolescents for each sex.

A comparative analysis between the nose-breathing group and the mouth-breathing group, based on the analysis of variance and Levene’s test7 at p<0.001, revealed different means. We therefore applied Dunnett’s test, 7 enabling us to compare the mean facial indices; we found no frequency differences between facial types (Table 5).

Our results agree with those of Linder Aronson (1970),18 who found a low frequency of adenoid patients with an increased facial height (25.9%).

These findings are different from those published by authors that used different methods, such as Mocellin & Ciuff (1997)1 and Hartgerink, Vig (1989).^12^ Their results suggested that mouth breathing influenced facial form in 58.4% of their patients that were labially incompetent, and 56.2% of their patients that were labially competent. Manganello, Silva and Aguiar (2002)^14^ found that 40% of mouth breathers had an increased facial height. Principato (1986)19 concluded that 67% of his orthodontic patients had an abnormal growth of the lower facial height and increased nasal resistance to the passage of air.

Our findings are also different from those of Jabur et al. (1997),15 who found an increased frequency of patients with a vertical growth pattern and mouth breathing (9 subjects - (39.13%) and 10 patients (43.47%) with a harmonic pattern.

## FINAL COMMENTS

Based on our findings we were unable to demonstrate a relation between mouth breathing and increased facial height. We thus agree with Bhat & Enlow (1995)^20^ and Kluemper (1995)^21^ who in their papers showed that there is individual, genetic and regional variation.

There is much controversy about whether mouth breathing leads to the long face syndrome. Many clinicians believe that delayed growth of the dentofacial complex is the result of environmental and genetic forces. Recent findings suggest that mouth breathing by itself is not necessarily harmful for growth. Some hereditary patterns may favor mouth breathing compared to others. Mouth breathing in a subject with a genetic propensity for excessive vertical facial growth may be a complicating factor for developing undesirable forms of malocclusion. Current literature and longitudinal studies on respiratory function and the development of the craniofacial complex are being reviewed. Some studies show little evidence that altered breathing would affect craniofacial morphology.

Further studies are needed in this area of knowledge.

## CONCLUSION

- We found no relation between mouth breathing and facial type.
- We found no statistically significant difference in the frequency of facial types in nose and mouth breathers.
- The mesoprosopic type was the most frequent facial type found in the current study; it was present in 25 adolescents (21.01%) of the mouth-breathing group, and in 23 adolescents (19.32%) of the nose-breathing group.
